# 1835. Factors impact Longevity in Persons Living with HIV

**DOI:** 10.1093/ofid/ofad500.1664

**Published:** 2023-11-27

**Authors:** Arshpal Gill, Cassandra Oehler, Srivastava Kodavatiganti, Samantha Baker, Chiu-Bin Hsiao

**Affiliations:** Allegheny General Hospital/Allegheny Health Network, Pittsburgh, Pennsylvania; Allegheny General Hospital, Positive Health Clinic, Center for Inclusion Health, AHN, Drexel University College of Medicine, Pittsburgh, Pennsylvania; Allegheny Health Network, Pittsburgh, Pennsylvania; Allegheny Health Network, Pittsburgh, Pennsylvania; Allegheny General Hospital, Positive Health clinic, Center for Inclusion Health, AHN; Drexel University, College of Medicine, Pittsburgh, Pennsylvania

## Abstract

**Background:**

In contrast to high mortality including young patient in 1990’s, persons living with HIV (PLWHIV) can expect a near-normal life expectancy of 77 years. Recent mortality data suggests significant number of HIV positive patients pass before the age of 70 years. There is a current paucity of literature looking at characteristics of the elderly PLWHIV when comparing to patients who died from HIV at a younger age. The goal of this study was to compare factors between the current HIV population living longer than age 70 and the population that did not survive until age 70 at the Positive Health Clinic (PHC). PHC is an urban city Ryan White funded HIV Care Medical Home Clinic, at Pittsburgh, PA, that provides care to ∼920 PLWHIV

**Methods:**

A retrospective chart review of patient records at PHC; with patients from period between 2015 -2022 was queried for HIV viral load, recent CD4 counts, and social history. Smoking, alcohol, and drug use were used as primary social measures due to their known impact in early development of prevalent chronic comorbidities. A Chi squared test was then employed to analyze for statistical significance of these factors (P < 0.05) between the cohort of patients that are living beyond the age of 70 (longevity) and the cohort of patients who died prior to age of 70

**Results:**

A total of 149 records were obtained (122M/ 27F, 95W/54B). The median age of HIV diagnosis and death was 44 and 60, respectively. There was no statistical difference between two groups in gender, race, and initial HIV transmission route, but being diagnosed with HIV after the age of 40 was associated with longevity. Unsurprisingly, patient with last viral load above 500 or CD4 below 200 were less likely to survive past age 70. HIV viral load < 200, persistently, in past 5 years and absence of smoking in last 15 years were the 2 factors most strongly associated longevity. Alcohol abuse and drug use were less likely to survive age 70. Statistical data between the two groups are listed in Table attached.

**Figure:**
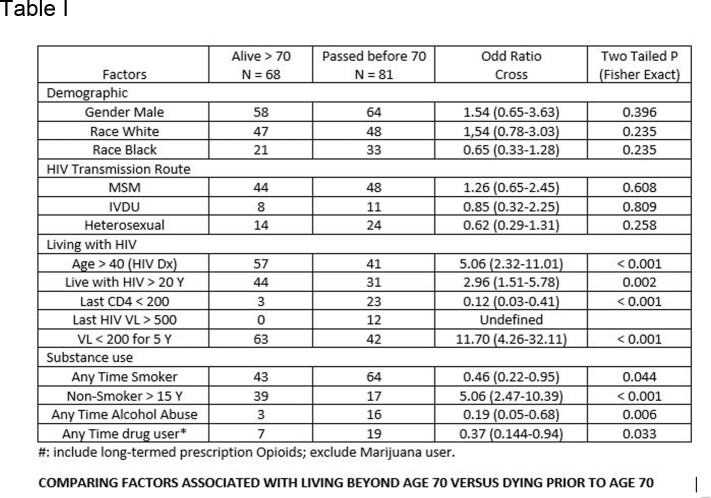

Comparing factors associated with living beyond 70 versus dying before 70

**Conclusion:**

Common comorbidities such as diseases of heart, lung, kidney and cancer are impacted by substance misuse, and as a result may serve as a barrier to reaching old age in PLWHIV. Counseling against tobacco use, treatment of alcohol and drug addiction while sustaining viral control with HAART can promote the longevity of PLWHIV.

**Disclosures:**

**Chiu-bin hsiao, MD**, Gilead: Grant/Research Support|ViiV: Advisor/Consultant|ViiV: Grant/Research Support|ViiV: Honoraria

